# Estimating the Burden of Febrile Illnesses

**DOI:** 10.1371/journal.pntd.0004040

**Published:** 2015-12-03

**Authors:** John A. Crump, Martyn D. Kirk

**Affiliations:** 1 Centre for International Health, University of Otago, Dunedin, New Zealand; 2 National Centre for Epidemiology and Population Health, Research School of Population Health, The Australian National University, Canberra, Australia; University of Zurich, SWITZERLAND

## Background

Fever without localizing features, hereafter referred to as fever or febrile illness, is among the most common reasons for persons in low-resource areas to seek health care [1,2]. The non-specific clinical presentation of many infections that cause fever makes it difficult to distinguish one from another based on clinical history and physical examination alone. Beyond malaria diagnostics, laboratory assays for many febrile diseases are often complex, costly, and may have limitations of sensitivity and specificity. Consequently, they are not widely available in low-resource areas where epidemiologic information on the etiology of febrile illness is sparse.

Undifferentiated fever is the main clinical feature of many diseases of global importance, including malaria, invasive bacterial diseases, several bacterial zoonoses, and many viral infections [3]. The World Health Organization (WHO) Foodborne Diseases Epidemiology Reference Group (FERG) is tasked with estimating burden of disease for conditions transmitted by food [4]. The vast majority of enteric conditions transmitted by food are associated with diarrhea, a syndromic grouping or “envelope” that can in turn be broken down by diarrhea-associated pathogen. However, a number of foodborne diseases are associated with fever rather than diarrhea, and the absence of a febrile illness “envelope” requires novel approaches to burden of disease estimation. Examples of such foodborne diseases presenting predominantly as febrile illnesses include typhoid and paratyphoid fevers, invasive non-typhoidal *Salmonella* disease, brucellosis, and listeriosis. It is likely that food safety interventions could have a substantial impact on the global burden of febrile illness [5]. Here, we describe some of the challenges and potential solutions to estimating burden of febrile conditions, including those transmitted by contaminated food.

## Challenges for Febrile Burden of Disease Estimation

**Fig 1:**
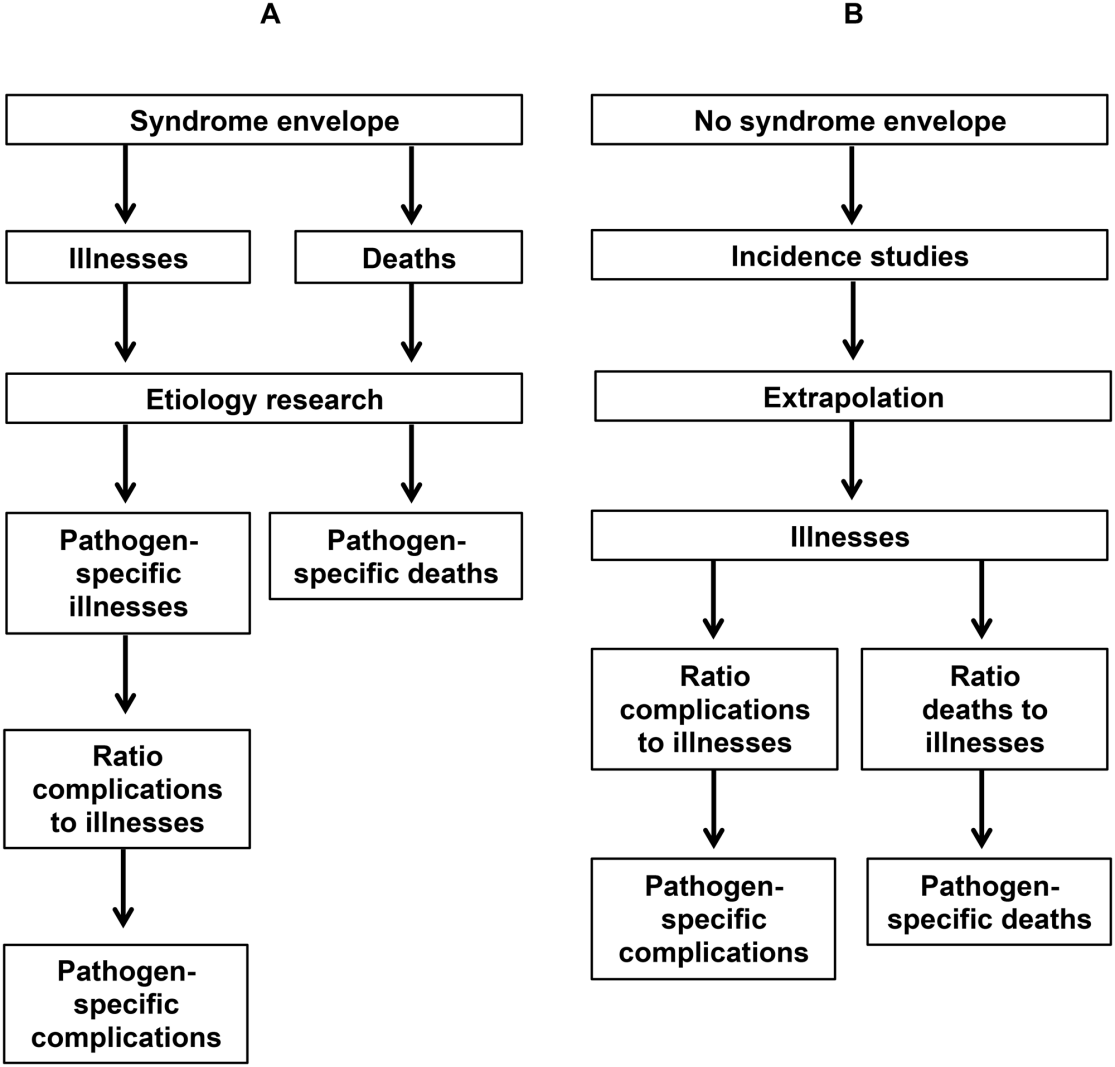
Approaches to estimating illnesses, complications, and deaths due to conditions with (panel A) and without (panel B) syndrome “envelopes.”

Burden of disease estimate tables for infections are structured by syndrome for some conditions and by disease group for others. For example, the burden of diarrheal diseases and lower respiratory tract infections are estimated first at the syndrome level by systematic review of studies measuring illnesses and deaths associated with each syndrome. In a second step, the burden of disease”envelope” for the syndrome is assigned to specific pathogens following systematic review of studies investigating the etiology of illness among persons with the syndrome of interest [4]. As such, the “envelope” of illnesses and deaths for diarrhea is assigned to cholera, *Salmonella*, *Shigella*, and so on. Complications may be estimated by deriving a ratio of complications to illnesses from further systematic review (Fig 1A). By contrast, the structure of burden of disease estimates does not include the syndrome of fever without localizing features. Pathogens causing febrile illness are instead represented as individual conditions (e.g., malaria, typhoid, and paratyphoid fevers), placed in groupings such as neglected tropical diseases, or do not feature at all (e.g., invasive non-typhoidal *Salmonella* disease, brucellosis, leptospirosis) and are presumably aggregated unnamed in “other” disease categories or uncounted.

## Designing an Approach to Estimating Febrile Illness Burden

In the absence of a disease burden”envelope” for the syndrome of fever, an alternative approach is to systematically identify pathogen-specific studies of disease incidence, complications, and deaths (Fig 1B). This approach poses a number of challenges and potential solutions, outlined below.

## Estimating Incidence

To accurately estimate disease incidence, studies require a population-based design with complete capture of cases, or an accurate estimate of under-ascertainment and laboratory confirmation of cases, to avoid the misclassification of conditions that cannot be reliably distinguished clinically. Because conducting such research is time-consuming and expensive, eligible incidence studies are rare, particularly for under-resourced or neglected diseases. Furthermore, designing incidence studies may be particularly challenging in urban areas with a range of types and levels of health care facilities [6,7]. To estimate incidence, data from a small number of studies that may lack representativeness must often be extrapolated to a much wider population.

## Approaches to Extrapolation

Extrapolation of incidence studies to other areas or population segments requires a rational basis and is clearly subject to considerable uncertainty that should ideally be explored by sensitivity analysis and represented with confidence intervals. Extrapolation of incidence data from one geographic area to another may be accomplished in a range of ways and with varying levels of sophistication. Sometimes extrapolation is done solely on the basis of geographic proximity, for example, within a United Nations area or region. The World Bank’s low-, lower-middle-, upper-middle-, and high-income groups take into account a measure of socioeconomic conditions that may be relevant for some diseases. A combination of regions and World Bank income categories may be used to account for both geography and socioeconomic conditions. Here, WHO member states are grouped into low- and middle-income countries (LMIC) by the six regions, separating out high-income countries within each of these regions into a seventh group.

Recognizing that disease risk may not be completely explained by either geographic proximity or socioeconomic conditions, a range of potential national or subnational characteristics may be studied by principal component analysis to form a basis for extrapolation [8]. For example, consumption of microbiologically unsafe water is a recognized risk factor for typhoid fever. Some groups have used national measures of access to improved water sources as a surrogate for microbiologically safe water to extrapolate typhoid fever risk between countries [9,10]. Similarly, recent malaria and HIV infection are recognized risk factors for invasive, non-typhoidal *Salmonella* disease [11]. Data from a small number of population-based studies of invasive non-typhoidal *Salmonella* disease have been extrapolated from one country to another using national measures of malaria prevalence and HIV seroprevalence [12].

Studies of disease incidence are often conducted in limited age groups, such as children under the age of 5 years. In such circumstances, it is necessary to extrapolate incidence in one age group to others. To do so, a robust understanding of the relationship between disease incidence and age is needed. Age-specific incidence is best understood from studies with active, population-based designs that have approximately equal sensitivity for case detection in children as in adults. For example, age-specific incidence of typhoid fever appears to be linked to the force of infection, with a large proportion of cases occurring among infants and young children in high-incidence settings, but affecting children and adults similarly in low-incidence settings [13]. By contrast, the incidence of non-typhoidal *Salmonella* disease among infants and young children is closely associated with malaria transmission intensity, and countries with generalized HIV epidemics show a second peak among young adults, reflecting a population immunocompromised by HIV infection [12,14].

## Accounting for Under-Ascertainment and Misclassification

Under-ascertainment of cases in disease incidence studies may be a function of the surveillance study design or related to the sensitivity of the laboratory assay used to confirm cases. Similarly, misclassification of cases may occur if a laboratory-based case definition is not used, or if the laboratory assay used to confirm cases has less than 100% specificity.

Under-ascertainment from study design may occur in any study that does not actively seek cases at the population level. For many febrile illnesses, under-ascertainment may occur in studies that do not seek cases by household visit several times per week and confirm cases at the household level. Due to the resource implications of household-based study designs, under-ascertainment is a common challenge. Under-ascertainment by study design may be addressed by measuring the proportion of cases not captured at each step of the study design and applying multipliers to account for such attrition [15]. For example, if typhoid fever surveillance is conducted at a sentinel health care facility, multipliers must be developed to account for the proportion of people who develop prolonged fever but do not seek care at the facility (e.g., by health care facility utilization survey) and the proportion of people with prolonged fever seen at the health care facility who do not receive blood culture (e.g., by monitoring the number of admissions with the syndrome and the number of blood cultures).

Ascertainment of cases by clinical history and physical examination alone poses a major risk for misclassification among febrile illnesses and should not be used. However, when confirming cases by laboratory assay, a clear understanding of the performance characteristics of the diagnostic test is needed and should be accounted for in estimates. For example, blood culture has approximately 50% sensitivity for the diagnosis of typhoid fever, yet blood culture is the diagnostic test most commonly used in robust typhoid fever incidence studies [16]. Therefore, cases detected by a single blood culture are doubled to account for under-ascertainment due to the sensitivity of the test. Furthermore, the sensitivity of blood culture is influenced by the volume of blood cultured, prior use of antimicrobials, and contamination by skin flora. Consequently, blood culture volume and contamination should be tightly controlled, and, when possible, blood cultures should be collected prior to administration of antimicrobials. In some circumstances, study designs might use tests with shortcomings of both sensitivity and specificity. Misclassification due to false-positive results may be accounted for by discounting incidence estimates or by enrolling a control group to allow estimation of attributable fraction [17,18].

## Estimating Complications and Deaths

Population-based studies of disease incidence are rarely large enough to accurately estimate the prevalence of pathogen-specific complications (e.g., intestinal perforation in enteric fever) and deaths. Outcomes may be modified by the early detection and correct diagnosis of cases inherent and appropriate in high-quality studies. Hospital-based studies are likely to be biased toward more severe cases or those with the ability to access hospital-level health care [15]. Furthermore, verbal autopsy is known to misclassify febrile deaths as due to malaria [19,20].

As a result, the ratio of complications to incidence and deaths to incidence are often estimated by expert opinion. Expert opinion may lack validity for a number of reasons, including, for example, being colored by the experiences of clinicians who work predominantly in health care facilities and see more severe cases. The risk for complications and death in typhoid fever is associated with the timeliness of antimicrobial therapy and the extent to which antimicrobial therapy matches the susceptibility of the infection organisms [21]. Both of these may vary in place and time.

## Way Forward

Many of the challenges for estimating the burden of febrile illnesses (Table 1) could be addressed by reorganizing diseases presenting predominantly with fever into a syndrome “envelope” of febrile illness. As is currently done for diarrheal diseases and lower respiratory tract infections, this “envelope” of disability-adjusted life years and deaths could then be assigned to specific pathogens on the basis of rigorous febrile illness etiology research [22,23].

**Table 1:** Key challenges and potential solutions for improving global burden of disease estimates for febrile illnesses.

Challenge	Potential solution
Lack of a burden of disease “envelope” for fever without localizing features	Conduct research to estimate the size of the burden of disease “envelope” for febrile illness
	Restructure burden of disease tables to include a syndrome”envelope” for fever under which all pathogens causing predominantly fever without localizing signs fall
Few robust studies of disease incidence, complications, and death	Develop standardized, population-based, multicenter fever etiology research prioritizing treatable and preventable infections
	Measure and account for under-ascertainment of cases from health care facility-based surveillance by studying patterns of health care utilization in catchment areas
	Promote research that improves the precision of estimates of the ratio of complications to incidence and deaths to incidence
Uncertainties for extrapolation of disease incidence, complications, and death to other areas, population segments, and age groups	Refine rational approaches to extrapolation, taking into account geographic proximity, socioeconomic conditions, and recognized risk factors for specific infections
	Study and adjust for the relationship between age and disease incidence, complications, and death
Non-specific clinical presentation of febrile illnesses and deaths	Use case definitions based on accurate laboratory assays that are standardized across studies
	Rely increasingly on pathologic autopsy rather than verbal autopsy for attribution of febrile deaths
Laboratory assays for some key infections lack sensitivity, specificity, or both	Test both case patients and healthy community controls to account for lack of test specificity and to calculate pathogen-specific attributable fraction
	Understand test performance against “gold standard” and adjust for lack of test sensitivity
	Improve the accuracy and versatility of diagnostic tests for febrile illnesses

Studies of fever etiology have tended to focus on just one pathogen (e.g., malaria) or use just one diagnostic test (e.g., blood culture). There are few fever etiology studies that have attempted to be comprehensive with respect to pathogens sought [22,23]. Fever etiology research that investigates a wide range of treatment and preventable pathogens in diverse geographies is needed to develop pathogen-specific etiology fractions to febrile illness [24]. Such an approach has been modeled for etiology research for severe childhood diarrhea [18] and pneumonia [17].

In the absence of a burden of disease “envelope” for fever without localizing features, approaches to estimate the incidence of specific infections are needed. Population-based surveillance with laboratory-based case definitions, active case finding, and a clear understanding of the denominator population are the conventional means of estimating incidence. However, such studies are expensive and time-consuming. Alternatives include sentinel health facility-based surveillance with “multipliers” derived from health care utilization studies [6,7,15] and vaccine probe studies for pathogens with available vaccines [25].

Innovative approaches are needed to gain a more accurate understanding of the complications-to-incidence ratio and the deaths-to-incidence ratio for febrile illnesses. It is unlikely that the specificity of verbal autopsy can be improved for febrile deaths. However, it is possible that pathologic autopsy could be made more widely available in low-resource areas to better classify febrile deaths in well-defined catchment populations [26].
